# Supplementary material for: In-depth investigation of genome to refine QTL positions for spontaneous sex-reversal in XX rainbow trout
Source: PLoS One. 2025 May 7;20(5):e0313464. doi: 10.1371/journal.pone.0313464 (PMC12058032; doi:10.1371/journal.pone.0313464)
Supplement: S1 Appendix — Positional candidate genes on Omy12 and Omy20 without known evidence of a functional role linked to sex-reversal (DOCX) [file pone.0313464.s001.docx]

**PLoS ONE**

In-depth investigation of genome to refine QTL positions for spontaneous sex-reversal in XX rainbow trout

A. Dehaullon†, C. Fraslin†, A. Bestin, C. Poncet, Y. Guiguen, E. Quillet, F. Phocas*

† Co-first authors: Audrey Dehaullon and Clémence Fraslin

*corresponding author’s email: florence.phocas@inrae.fr;

**S1 Appendix. Positional candidate genes on Omy12 and Omy20 without known evidence of a functional role linked to sex-reversal**

***hcn1* as the best positional candidate for QTL on Omy12**

Despite of its lesser importance to explain sex-reversal, the QTL on Omy12 appears to be primarily associated to *hcn1* (Hyperpolarization Activated Cyclic Nucleotide Gated Potassium Channel 1) gene both in the discovery population and in some validation ones. hcn1 is a non-selective cation channel that is expressed in retina, brain and heart (see S1 Online Resource, S9 Table). As far as we know, there is no link identified between variants in this gene and sex-reversal phenotype, but it has been associated with a reproductive behavior in human, the age at first sexual intercourse [1]. In addition, it is a gene playing an important role in the general stress response as shown in Atlantic salmon for hyperthermia stress where the induction of hcn1 expression in the liver cells may be important in maintaining/elevating mitochondrial energy metabolism under heat stress [2].

***akt3*, *cep170aa*, *sdccag8* as additional candidate genes for Omy20_a**

***Implication of akt in the phosphoinositide 3-kinase (PI3-K) signaling pathway***

As reviewed by Cecconi et al. [3], the phosphoinositide 3-kinase (PI3-K) signaling pathway plays an important role in regulating fundamental cellular processes such as cell growth, survival, proliferation, and metabolism, largely mediated via the protein kinase B/v-akt murine thymoma viral oncogene homolog (*akt*) signaling. akt3 is one of 3 closely related serine/threonine-protein kinases (*akt1*, *akt2* and *akt3*), which regulate many processes including metabolism, proliferation, cell survival, growth and angiogenesis, in response to insulin and growth factors. They are involved in a wide variety of biological processes including cell proliferation, differentiation, apoptosis, tumorigenesis, as well as glycogen synthesis and glucose uptake. *akt3* is the least studied *akt* kinase. It plays an important role in brain development. One intronic SNP in *akt3* was significantly detected as playing a role in sex-reversal in the discovery population (Table 4) as well as a haplotype including this gene was among the 10 best ranked in the full RF analysis accounting for all QTL regions (S8 Table). An important paralog of *akt3* is *akt1* (also named *akt*). The Pi3k/Akt signaling pathway has been implicated in stem cell maintenance, organogenesis and primordial follicle activation [4]. In the mammalian ovary, *akt* determines the pool of primordial follicles and the transition from quiescent to growing phase. In addition, it modulates granulosa cell apoptosis throughout folliculogenesis. In oocytes *akt* participates in the control of meiosis resumption and, at metaphase II stage, regulates polar body emission and spindle organization [5]. Pi3k/Akt pathway induces Pdk1/Akt/S6k1/rpS6 signaling in primordial follicle oocytes [6]. In vitro experiments also demonstrated that cold stress inhibited the expression of *akt*, *pi3k*, and *s6k* in fish *C. bouderius*, with associated effects on the interplay between autophagy and apoptosis [7]. It remains to know whether or not akt3 play similar roles than *akt1* in cell apoptosis throughout folliculogenesis.

***cep170aa and sdccag8 as putative candidates interacting with cilk1 in OMy20_b?***

The protein encoded by *cep170aa* (centrosomal protein 170Aa) localizes to the sub-distal appendages of mature centrioles, which are microtubule-based structures thought to help organize centrosomes. During mitosis, the protein associates with spindle microtubules near the centrosomes. Among its related pathways is Ciliary landscape (S9 Table). Two intronic SNPs were among the 100 (or even 50) best ones in DAPC and another SNP had a p-value ≤ 0.002 (Table 4) in the discovery population. No information is directly related to sex differentiation for this *cep170aa* gene.

*sdccag8* (SHH signaling and ciliogenesis regulator) is a centrosomal/basal body protein essential for proper cilia formation. Only two SNPs have been identifed with p-value ≤ 0.002 (Table 4), while no haplotype in RF analysis neither single marker in DAPC were selected as important to explain sex-reversal in our discovery population. Gene mutations in *sdccag8* have been found in patients with ciliopathies manifesting a broad spectrum of symptoms, including hypogonadism. Among these mutations, several that are predicted to truncate the *sdccag8* carboxyl (C) terminus are also associated with the same symptoms; Tsutsumi et al. [8] identified the Sdccag8 C-terminal region (Sdccag8-C) as a module that interacts with the ciliopathy proteins, *cilk1* and *mak*, which were previously shown to be essential for the regulation of ciliary protein trafficking and cilia length in mammals. In mutant mice in which Sdccag8-C was truncated, abnormalities were observed in cilia formation and ciliopathy-like organ phenotypes as well as a defect in spermatogenesis [8].

***cilk1* and *rps6ka2* as additional gene candidates for QTL Omy20_b**

***Potential implication of cilk1* *in sexual differentiation in vertebrates***

*cilk1* (serine/threonine-protein kinase ICK) has an essential role in human development. It regulates the ciliary localization of *shh* pathway components, the intraflagellar transport (IFT) speed, and negatively regulates cilium length in a cAMP and mTORC1 signaling-dependent manner. It may play a key role in the development of multiple organ systems (see S1 Online Resource, S9 Table). In our study, a haplotype included *cilk1* was among the 20 best ranked in the full RF analysis (see S1 Online Resource, S8 Table) and 5 SNPs were among the 100 best in DAPC, 3 being upstream intergenic, while one was a synonymous mutation at position 34,620,738 bp and a last SNP was intronic (see S1 Online Resource, S10 Table).

The primary cilium is an organelle involved in cell signaling, which controls development of many organs, but the role of primary cillium in the sex determination and sexual differentiation of gonads is completely unknown. Piprek et al. [9] observed a high level of expression of numerous markers of primary cilium in fetal mouse gonads, indicating that the primary cilia and/or primary cilia-related genes may be important for development of both somatic and germ cells in the gonads. Primary cilia are signalling hubs for Hedgehog and Wnt signalling pathways, both of which play a major role in the development and regeneration of adrenal glands [10]. It is thus conceivable that *cilk1* may be involved in the development of adrenal glands through regulating various ciliary signalling pathways.

***Implication of rps6ka2 in maintaining primordial and developing follicles***

As regards *rps6ka2* (ribosomal protein S6 kinase alpha-2), it is a serine/threonine-protein kinase that acts downstream of ERK signaling and mediates mitogenic and stress-induced activation of transcription factors, regulates translation, and mediates cellular proliferation, survival, and differentiation. As reviewed by Sobinoff et al. [6], *rps6* in oocytes is important in maintaining the survival of primordial and developing follicles. *rps6* was shown to be subject to phosphorylation in response to multiple physiological and pathological stimuli, including hypoxia and osmolarity [11]. It is also linked to the Pi3k/Akt pathway described previously.

**References**

1. Day FR, Helgason H, Chasman DI, Rose LM, Loh PR, Scott RA, et al. Physical and neurobehavioral determinants of reproductive onset and success. Nat Genet. 2016 Jun;48(6):617-623. doi: 10.1038/ng.3551.
2. Beemelmanns A, Zanuzzo FS, Sandrelli RM, Rise ML, Gamperl AK. The Atlantic salmon's stress- and immune-related transcriptional responses to moderate hypoxia, an incremental temperature increase, and these challenges combined. G3 (Bethesda). 2021 Jul 14;11(7):jkab102. doi: 10.1093/g3journal/jkab102.
3. Cecconi S, Mauro A, Cellini V, Patacchiola F. The role of Akt signalling in the mammalian ovary. Int J Dev Biol. 2012;56(10-12):809-17. doi: 10.1387/ijdb.120146sc.
4. Cantley LC. The phosphoinositide 3-kinase pathway. Science. 2002 May 31;296(5573):1655-7. doi: 10.1126/science.296.5573.1655.
5. Sobinoff AP, Sutherland JM, Mclaughlin EA. Intracellular signalling during female gametogenesis. Mol Hum Reprod. 2013 May;19(5):265-78. doi: 10.1093/molehr/gas065.
6. Reddy P, Adhikari D, Zheng W, Liang S, Hämäläinen T, Tohonen V, et al. PDK1 signaling in oocytes controls reproductive aging and lifespan by manipulating the survival of primordial follicles. Hum Mol Genet. 2009 Aug 1;18(15):2813-24. doi: 10.1093/hmg/ddp217.
7. Wang D, Tian Y, Wang Q, Zhang Y, Ye B, Zuo Z et al. Cold stress-induced autophagy and apoptosis disorders are mainly mediated by AMPK/PPAR/PI3K/AKT/mTOR pathways. Aquaculture. 2024;740574. doi: 10.1016/j.aquaculture.2024.740574.
8. Tsutsumi R, Chaya T, Tsujii T, Furukawa T. The carboxyl-terminal region of SDCCAG8 comprises a functional module essential for cilia formation as well as organ development and homeostasis. J Biol Chem. 2022 Mar;298(3):101686. doi: 10.1016/j.jbc.2022.101686.
9. Piprek RP, Podkowa D, Kloc M, Kubiak JZ. Expression of primary cilia-related genes in developing mouse gonads. Int J Dev Biol. 2019;63(11-12):615-621. doi: 10.1387/ijdb.190049rp.
10. Finco I, Lerario AM, Hammer GD. Sonic Hedgehog and WNT Signaling Promote Adrenal Gland Regeneration in Male Mice. Endocrinology. 2018 Feb 1;159(2):579-596. doi: 10.1210/en.2017-03061.
11. Meyuhas O, Dreazen A. Ribosomal protein S6 kinase from TOP mRNAs to cell size. Prog Mol Biol Transl Sci. 2009;90:109-53. doi: 10.1016/S1877-1173(09)90003-5.
