# Supplementary material for: In-depth investigation of genome to refine QTL positions for spontaneous sex-reversal in XX rainbow trout
Source: PLoS One. 2025 May 7;20(5):e0313464. doi: 10.1371/journal.pone.0313464 (PMC12058032; doi:10.1371/journal.pone.0313464)
Supplement: Attachment — Submitted filename: Neobio_response_to_reviewers_PlosOne.docx [file pone.0313464.s004.docx]

Dear Editor and reviewers,

We would like to thank you and the three reviewers for your positive’s comments on our manuscript.

Please find attached the revised version of our manuscript “**In-depth investigation of genome to refine QTL positions for spontaneous sex-reversal in XX rainbow trout**”. The manuscript has been revised according to all the suggestions given by the reviewers. We thank the three reviewers for their valuable comments and suggestions that, we believe, considerably improved the clarity of the manuscript.

One of the main comments from reviewers and the editor was the length of the manuscript, especially the discussion and introduction. We did our best to shorten those two sections by removing details, however we believe that there is a lot of interesting points to mention in the discussion that we have kept. We also broadened the discussion by adding a section on the importance of the spontaneous masculinization in ecological context as requested by R#2 and the editor. Thus we have not been able to reduce the discussion as much as we would have wanted. Yet we hope that the effort we made are sufficient and that this manuscript now meets PLOS ONE’s publication criteria and you will consider it for publication.

In addition, for the role of funders, we declare that the funders had no role in our study. Therefore, the statement to be changed on our behalf on the online submission form is: “The European Maritime and Fisheries Fund and the French National Government supported the NeoBio project (R FEA470016FA1000008). The funders had no role in study design, data collection and analysis, decision to publish, or preparation of the manuscript."

Below, we provide the detailed responses to the reviewer's comments. We started with the main 5 points you, the editor, raised as main issues and have then numbered the reviewers’ comments to facilitate further discussion. All the changes made in the manuscript are displayed as red highlights in the track changes version.

Yours sincerely,

Dr Clémence Fraslin, Audrey Dehaullon and Dr Florence Phocas

------------------------------------------

The manuscript represents high quality work of considerable interest to the readers of PlosOne. The concerns of the reviewers and editors centre on flow and writing, not design or analyses. The main issues are the following.

1. Provide details on bioinformatic analyses and check on comments about analyses by reviewer 1.

We added details on the bioinformatics analyses, mainly the variant calling and quality controls. The detailed answer to Reviewer 1 is available under their point 3.

2. Provide better flow (thread) through the manuscript.

We have now revised the introduction and the discussion to shorten it, hoping it will improve the flow of the manuscript. We also tried to better explain what we intend to do in the M&M section by adding a few points in the introduction of each sub-section as suggested by R1 as well as adding a new Figure 1 to better follow the process of the different analyses.

3. Rev 2 asks for a broadened discussion of the spontaneous masculinization, in ecological context (can shift balance in discussion towards this point).
4. Related to that (rev2) the interaction of rearing temp and genetic factors, is interesting beyond students of aquaculture. Feel free to integrate these ideas into the discussion, again by removing or condensing other points in that section.

We added a paragraph on this point (masculinisation in an ecological context and impact of the rearing temperature in both farm and ecological context) the end of the discussion section before a shorten conclusion. We added 4 new references to support that discussion. The details of the new paragraph are given in response to reviewer 2 point 2. Since little is known of spontaneous masculinisation of wild rainbow trout and the impact of high temperature on masculinisation is still being investigated, we did not change the focus of the discussion towards this point.

We added four new references to support our discussion.

100. Valdivieso, A., Wilson, C. A., Amores, A., da Silva Rodrigues, M., Nóbrega, R. H., Ribas, L., et al. Environmentally-induced sex reversal in fish with chromosomal vs. polygenic sex determination. Environmental research. 2022 Oct; 213, 113549. doi:10.1016/j.envres.2022.113549
101. Cavileer TD, Hunter SS, Olsen J, Wenburg J, Nagler JJA. Sex-Determining Gene (sdY) assay shows discordance between phenotypic and genotypic sex in wild populations of chinook salmon. Transactions of the American Fisheries Society. 2015 March; 144:423-430. doi: 10.1080/00028487.2014.993479
102. Bezault, E., Clota, F., Derivaz, M., Chevassus, B., & Baroiller, J. F. Sex determination and temperature-induced sex differentiation in three natural populations of Nile tilapia (Oreochromis niloticus) adapted to extreme temperature conditions. Aquaculture. 2007; 272, S3-S16. doi: 10.1016/j.aquaculture.2007.07.227
103. Shinomiya, A., Otake, H., Hamaguchi, S., & Sakaizumi, M. Inherited XX sex reversal originating from wild medaka populations. Heredity. 2010 April; 105(5), 443-448. doi: 10.1038/hdy.2010.51
5. The text needs to be shortened (rev 3), but some things also need clarifying (rev 1, mainly to do with analyses and validation of findings).

We added a figure to better follow the flow of the analyses. We renamed the sections in the methods and added a short introduction paragraph at the beginning of each methods following reviewer 1 recommendation and we believed that this improved the clarity of the manuscript.

We also tried to reduce the introduction and the discussion, hoping to shorten the manuscript. In the introduction, we only succeeded to gain 2 lines as we had to add the full names of genes as recommended by reviewer 3. As we added a new paragraph in the discussion (point 3 and 4 above), the number of lines for “Discussion & conclusion” went in fact from 306 to 307. As we also added details of the variant calling methods (point 1), the efforts made to reduce the initial contents of the introduction and discussion had thus no impact on the length of the manuscript. We are sorry for that, but feel that all the points that are introduced or discussed in the text are needed to help with the understanding of spontaneous masculinization.

We are addressing each reviewers’ comment in more details in the sections below. We included lines numbers from the tracked changes manuscript and reported in the response the additional text that is now included in the manuscript.

Reviewer #1: We thank Reviewer #1 for their appreciation of our manuscript. We think that the reviewer’s comments helped improve the clarity of the manuscript. We addressed R#1 first two main comments and other specific comments below.

1. “The methods employed in the manuscript are many, and as reader, I missed a logical thread thought the methods and results section. This could be achieved with a short introduction paragraph at the beginning of each section letting the reader know what type of information is expected from the analysis, and how it complements the analysis from the former section.”

We thank the reviewer for this useful comment, we have now added sentences in the methods to explain why the approach was used and we also added a graphical summary of the approaches and dataset used in a new **Fig 1**. We hope that this helped improve the clarity and the flow of the manuscript.

**“Fig. 1. Graphical summary of the different statistical analyses performed in the discovery or validation populations.”** (Lines 219-220)

“We sought to validate the existence of the four QTL detected by Fraslin et al. [27] as linked to spontaneous sex-reversal of XX trout in six diverse French populations of rainbow trout (Table 1, Fig. 1). Population A was composed of XX sibs from the same birth cohort of the parents used to produce the initial QTL discovery population [27]. Fish from populations B and C and those from populations E and F came from two other French breeding compagnies, while fish from population D came from a commercial site using fry from unknown origin.” (Lines 198-293)

“**Exact Fisher test on all SNPs of QTL in 23 extreme dams of the discovery population**

In order to refine the QTLs boundaries and confront the results between the validation and discovery populations (Fig. 1), an exact Fisher test was run to detect the association of all the sequence variants observed in the four extended QTL regions with the average progeny sex-ratios of the 23 extreme dams of the discovery population. ” (Lines 234-238)

“To refine the QTL locations in the discovery population using the latest genome assembly we i) used Random Forests approaches with haplotypes and ii) applied a Discriminant Analysis of Principal Components on the sequence data from the 23 dams selected above.” (Lines 247-249)

2. “The word “validation” is used along the manuscript to describe different things, this has a potential to confuse the reader. There is a general aim to validate the QTLs from Fraslin et al. There is also an internal validation dataset within random forest. And finally, from my understanding you used a separate discovery and validation population for the Fisher test, however this remains to be clarified. I found it difficult to grab how the loci detected in the discovery population were matched in the validation group. The only mention of it is in Line 219, but I found it unclear.”

Indeed, we used “validation” to refer to many things in the original manuscript. We made an effort to modify the text to make it clearer.

We are now using “validation” population and “validate” the QTL only when we talk about the “six validation populations” that are distinct from the original discovery population from Fraslin et al [27]. And we use “refine” or “confirm” to describe the new QTL position in the discovery population after mapping to the new genome reference (Arlee). We hope that the new Figure 1 will help clarify that point as well.

Regarding the reviewer’s comment on the validation of loci detected in the discovery population: we rephrased the methods used for the genotyping of the validation population using 96 SNPs arrays containing SNPs identified in the discovery population by Fraslin et al [27].

“Two 96 SNPs genotyping arrays using microfluidic real-time PCR Fluidigm Kasp chemistry were designed. A set of 192 SNPs identified in Fraslin et al [27] (see S1 Supplementary Tables, S2 Table) were selected, corresponding to: 140 SNPs in the two main QTL identified on chromosome Omy1, 19 SNPs in a putative QTL region detected on Omy12 and 33 SNPs in a large 6 Mb region with putative QTL on Omy20.

Pieces of caudal fin sampled from 315 fish, corresponding to at least 30 XX fish per population, including a minimum of eight neomales (Table 1), were sent to Gentyane genotyping platform (INRAE, Clermont-Ferrand, France) for genotyping after DNA extraction using the DNA advance kit from Beckman Coulter following manufacturer instructions.

After quality control, 19 SNPs were eliminated (including 14 from Omy1) from the analyses of all the populations because their genotyping rate was below 90%, 173 SNPs were kept for validation of the QTL.” (Line 198-215)

The methods to validate the relevance of those SNPs using the exact Fisher test are described lines 222 to 244. We added a new header to the section in the methods where we describe another exact Fisher test performed, this time, in the discovery population:” **Exact Fisher test on all SNPs of QTL in 23 extreme dams of the discovery population”**. We think that the section below is the one the reviewer refers to (previously lines 218-225), we modified this section to make it clearer what test is performed in the discovery population.

**Specific comments**

3. Line 165-176. Here you skip many details about the sequence mapping, SNP calling and SNP filtering methods. I think that the exact parameters used in GATK, SAMtools etc should be given in the method section. In addition, the number of SNPs/indel reported here seems very high (about one for every 80bp if we consider the USDA_OmykA_1.1 genome is 2.3Gb long). This number is not completely surprising for a brut output from Mpileup, however the 21.10^6 SNP remaining after filtering (still one for every 110 bp), sounds like a very high density of good quality SNPs. I would therefore like to have a description of the parameters/ criterion that were used for filtering the SNPs as well as some basic statistics from the mapping (mean coverage ±sd).

In Gao et al (2018) and Bernard et al. (2022) you will see that the rainbow trout genome is extremely polymorph. In Bernard et al. (2022), the average distance between two successive high-quality variants was 60 bp on the Swanson rainbow trout reference genome, consistent with the value of one SNP every 64 bp previously reported by Gao et al. (2018).

Gao, G., Nome, T., Pearse, D. E., Moen, T., Naish, K. A., Thorgaard, G. H., et al. (2018). A New Single Nucleotide Polymorphism Database for Rainbow Trout Generated Through Whole Genome Resequencing. *Front. Genet.* 9, 147. doi:10.3389/fgene.2018.00147.

Bernard M., Dehaullon A., Gao G., Paul K., Lagarde H., Charles M., Prchal M., Danon J., Jaffrelo L., Poncet C., Patrice P., Haffray P., Quillet E., Dupont-Nivet M., Palti Y., Lallias D., Phocas F. (2022). Development of a High-Density 665 K SNP Array for Rainbow Trout Genome-Wide Genotyping. Frontiers in Genetics, 13, 941340 <https://dx.doi.org/10.3389/fgene.2022.941340>

We have now added more details on the new mapping as well as for the variant calling and filtering in methods and the section now reads:

“On average, 9.252 10^7^ paired reads (1.456 10^7^ sd) were mapped on the Arlee genome, corresponding to a mean coverage of 12X. While the percentage of properly paired sequence reads over all sequenced pairs was in average 87.3% for the alignment against Swanson’s genome assembly, these statistics went up to 94.1% for the alignment against Arlee’s genome assembly.

After mapping, duplicates were marked using the MarkDuplicate tool, and base quality score where recalibrated using ApplyBQSR tool from GATK 4.2.2.0 [32], then variants were called independently with the three different variants calling tools HaplotypeCaller from GATK, Freebayes 1.3.5 [33] and SAMtools Mpileup 1.11 [34]. Recommended quality filters were used to ignore low-confidence alignments: a minimum mapping quality of 30, a minimum base quality required to consider a base for calling of 10X reads, minimum phred-scaled confidence threshold at which variants should be called of 30. A total of 29,229,949 variants that were obtained by the three different variant calling tools were kept for further quality controls using vcftools 1.15 and keeping indels and SNPs located only on known chromosomes, removing variants located on un-located contigs or mitochondrial chromosome. Further quality filtering on variant coverage were performed using SelectVariant and Variant Filtration tools (GATK) according to the hard-filtering recommendations. Briefly QD < 2.0 (to normalize variant quality), FS > 6.0 (measure of the Phred-scaled probability that there is strand bias at the site), MQ < 40.0 (mapping quality), SOR > 3.0 (Strand Odds Ratio), MQRankSum < -12.5 (u-based z-approximation from the Rank Sum Test for mapping qualities) and ReadPosRankSum < -8.0 (u-based z-approximation from the Rank Sum Test for site position within reads).” (Lines 166 - 186)

4. Regarding the analysis performed with the SNPs from full sequence data (random forest & DAPC), you only use the genomic regions reported previously by Fraslin et al, however, if I understand correctly, you have sequenced the full genome of the females. You could thus compare the distribution of the test between putative QTL region and non-QTL regions. This would provide with an empirical null distribution of the test (Gini index & allele contribution to discriminant axes).

It would have been difficult or impossible to use the random forest and DAPC approaches on the full genome or even the full chromosome (so including QTL and non-QTL region) due to the limited number of individuals (23 sequenced dams with extreme offspring distribution) we could consider in the analysis. As we mention (lines 386-388), we already had too many variables in the joint RF analysis of the 4 QTL regions, and probably created some « noise » by considering over 1,100 haplotypes together in the analysis to classify only 23 individuals. Our aim was not to have some test statistics when running the RF and DAPC analyses, but only to rank the more important haplotypes and SNPs to predict the correct classification.

5. Table3: I don’t understand the reason for splitting the Omy1 QTL into two where Omy1-a stops at 67,960,401 bp and Omy1-b starts at the next base 67,960,402. I also see that each QTL has one given position (based on the Arlee reference genome), but despite the table legend, I didn’t find any comparison between these QTL positions and the QTL reported by Fraslin et al.

We did not split the Omy1 QTL into as it was already clear that there were 2 close QTL regions in our previous study [27] with boundaries on Swanson genome references: 63 229 533 - 63 560 537 bp for Omy1_a, and 64 354 655 – 64 707 100 bp for Omy1_b. When we extended the boundaries for the two analyses we thus decided to completely study the 2 corresponding Mb on the Arlee genome.

To help with the correspondence between the two studies, the border of the initial QTL by Fraslin et al [27] where added in the legend of Table 3. Although we understand the reviewer comments, we decided not to include the previous boundaries in Table 3 itself to avoid confusion as they were obtained on a previous genome assembly.

“The initial four QTL boundaries were 63 229 533 - 63 560 537 bp, 64 354 655 – 64 707 100 bp, and 5 751 451 - 6 965 249 bp respectively for Omy1_a, Omy1_b, and Omy12 positioned on Swanson reference genome, and in a vague vicinity of 31 352 390 bp for QTL on Omy20.” (Lines 361-363)

We do not think it was appropriate to map the previous boundaries to the new assemble used in the current study as the differences between the two assemblies is in average 4Mb, but not at all constant along the genome. The positions on both assemblies of the 192 SNPs used for the validation are available in table S3 and this is now mentioned in the text lines 336-340: “given in S1 Supplementary Tables, S3 Table with indication of the level of significance in those populations, as well as the corresponding positions of SNPs on both reference genomes.”

6. Line 384: This could be reformulated. How did the present results make LOC110527930 a less good candidate and tlx1, fbxw4… better ones? Fig3 is a dapc plot, I am not sure how it illustrates the point. (Figure numbers might have been mixed in the text, please check)

The best-ranked haplotypes according to the random forest analysis were used to determine which genes are likely to have a stronger effect on masculinisation.

This was unclear and the previous sentence: “While LOC110527930 initially was our favourite causative gene for Omy1_b, genes such as tlx1, cep68, fbxw4 and hells are now preferred within Omy1_b region (Fig. 3).” has been rephrased to highlight this and now reads: “While LOC110527930 initially was our favourite causative gene for Omy1_b, the best-ranked haplotypes now point towards genes such as tlx1, cep68, fbxw4 and hells that are now preferred within Omy1_b region (Fig. 2).” (Lines 417-419).

The figure number has also been corrected is now Fig. 2 (new Fig. 1 added).

7. Line 419 ACP -->PCA

The French term was used in the original text and has now been corrected in the English version.

8. Line 419: What are “all those variants”? Is that the set of 33,731 SNPs? You use different subsets of data and analysis methods along the manuscript. It is thus a good idea to keep using precise wording and not hesitate to remind the reader which data you are currently commenting.

The sentence has been rephrased to indicate the number of variants in the analysis. “Running a simple PCA on all those 27,828 variants (without missing genotypes out of 33,731 SNPs) do not allow to correctly discriminate the two groups of dams as shown by Fig.4A.” (Lines 453-455).

We also took the reviewer’s comment here and main comment 1 in account and we tried to be clearer on which analysis was performed on what dataset. For this we also included a new Fig. 1 to summarize the different approaches and sub-datasets we used.

------------------------------------------

Reviewer #2:

The methodology used is particularly novel, and the large volume of data generated is a significant strength of the study. Genes such as syndig1, tlx1, and hells on Omy1, along with khdrbs2 and csmd1 on Omy20, have been highlighted for further functional studies, including their interaction with environmental factors like rearing temperature. These findings provide valuable insights for improving sex-ratio control and producing more consistent all-female populations, which are preferred in aquaculture due to their delayed maturation and reduced susceptibility to diseases.
The work is highly interesting and generates a substantial amount of useful information.

We thank the reviewer 2 for their appreciation of our manuscript and of the quality of our work and amount of information generated by his study.

I have a few minor comments for consideration:

1. The authors highlight that spontaneous masculinization is a highly heritable trait controlled by minor sex-modifier genes, with several QTL regions previously identified. While the importance of monosex populations in aquaculture is well established, the implications of this phenomenon in wild populations require further exploration. Even if spontaneous masculinization occurs at a low frequency in nature, its potential effects on population dynamics, genetic diversity, or ecological stability are worth investigating. Addressing these aspects would not only broaden the ecological context of the study but also underline the relevance of understanding this process beyond its aquaculture applications.

2. The conclusion highlights the challenge of undesirable masculinized progeny and the need to better understand the combined effects of genetic and environmental factors on spontaneous maleness in all-female stocks. Given the role of temperature in sex reversal, I suggest that future research could explore in more detail the interactions between genetics and rearing temperatures. Specifically, it would be interesting to consider how these factors might be controlled to achieve the desired all-female progeny, particularly when using free-hormone neomales. Additionally, it could be valuable to outline potential experimental approaches to assess the influence of rearing temperatures at different developmental stages. Exploring whether specific temperature thresholds or genetic combinations could minimize masculinization while optimizing production efficiency may also provide important insights. I believe that discussing these potential avenues for future research, based on the findings of this study, would help to further contextualize the results and expand their applicability to sex ratio management in aquaculture.

I trust that addressing these aspects will further strengthen the manuscript and provide additional perspectives on how to advance the field.

We took the reviewers comments and suggestion for a broader discussion in considerations and we now address spontaneous sex reversal in a farm and ecological context (point 1) and the effect of temperature and genetic by temperature interaction (point 2) in a new section in the discussion (see below and lines 746-773).

“**Interaction effects between spontaneous masculinization and rearing temperature in farming and ecological contexts**

In addition to this genetic control, it is well known that water temperature during development can modulate sex differentiation in many fish species and, in particular, high temperature is associated with masculinization [97]. In rainbow trout, high rearing temperature during the early phase of fry development after hatching was shown to increase the frequency of sex reversal in XX females to a greater or lesser extent depending on the genetic background of the trout lines [98, 99]. From a fish farmer perspective, further investigation is still needed before any operational use of the genotypes at minor sex-modifier genes either to eradicate spontaneous sex-reversal in all-female production stocks or to produce progeny from free-hormone neomales in rainbow trout. Indeed, the high heritability of maleness [27] suggests that the use of spontaneously masculinised individuals as progenitors of all-female stocks would increase the frequency of undesirable masculinised progeny. Improve knowledge about the joint effects of genetic basis and environmental factors determining spontaneous maleness in all-female stocks is then needed to search for a trade-off combining together genetic and environmental control of gonad masculinization according to the destination of the fish (broodstock vs all-female production stock).

From an ecological point of view, the question of the extent of spontaneous sex reversal arises as a key one for conservation biology and wild fish population sex ratios in a global warming context [100]. A study on zebrafish [100] compared the impact of elevated-temperature on sex reversal in different strains with chromosomal or polygenic GSD. They showed that strains with a major sex-determining locus may not be immune to temperature driven sex reversal. Spontaneous sex reversed females in chinook salmon (*O. tshawytscha*), Nile tilapia (*Oreochromis niloticus*), zebrafish (*Danio rerio*) and lake trout (*Salvelinus namaycush*), have been observed at low frequency in the wild [1, 22, 100, 101, 102, 103]. The impact of spontaneous masculinization in wild rainbow trout on population dynamics and fitness remains unknown and deserves more attention as this highly heritable phenomenon, driven by environmental factors, may become more frequent with warmer water temperature.”

We added four new references to support our discussion.

104. Valdivieso, A., Wilson, C. A., Amores, A., da Silva Rodrigues, M., Nóbrega, R. H., Ribas, L., et al. Environmentally-induced sex reversal in fish with chromosomal vs. polygenic sex determination. Environmental research. 2022 Oct; 213, 113549. doi:10.1016/j.envres.2022.113549
105. Cavileer TD, Hunter SS, Olsen J, Wenburg J, Nagler JJA. Sex-Determining Gene (sdY) assay shows discordance between phenotypic and genotypic sex in wild populations of chinook salmon. Transactions of the American Fisheries Society. 2015 March; 144:423-430. doi: 10.1080/00028487.2014.993479
106. Bezault, E., Clota, F., Derivaz, M., Chevassus, B., & Baroiller, J. F. Sex determination and temperature-induced sex differentiation in three natural populations of Nile tilapia (Oreochromis niloticus) adapted to extreme temperature conditions. Aquaculture. 2007; 272, S3-S16. doi: 10.1016/j.aquaculture.2007.07.227
107. Shinomiya, A., Otake, H., Hamaguchi, S., & Sakaizumi, M. Inherited XX sex reversal originating from wild medaka populations. Heredity. 2010 April; 105(5), 443-448. doi: 10.1038/hdy.2010.51

------------------------------------------------------------------------

**Reviewer #3, Martin Knytl:**

The proposed manuscript “In-depth investigation of genome to refine QTL positions for spontaneous sex-reversal in XX rainbow trout” significantly contributes to the study of evolutionary biology, particularly in understanding the evolution of sex determination and its pathways. The manuscript is technically sound and written in high-quality English. Impact of the study is well highlighted and the statistical analyses are appropriately chosen and used. It would be beneficial if the authors included Sanger sequencing results for selected SNPs, especially those that are 100% sex-linked. However, the manuscript is undoubtedly of high quality and meets the requirements for the Plos One journal. I believe it will also be of interest to readers of the journal. I have only minor suggestions:

We thank Dr Martin Knytl for his appreciation of the quality of our manuscript and statistical approached. We appreciated the comment on the interest to readers of the journal.

1) Manuscript is very long and requires the readers’ full intention. The introduction spans more than three pages, and the discussion is more than 9 pages long. I think this extensive length may diminish the reader's interest. Some parts are explained in great detail and I suggest shortening these sections where possible.

We agree with the reviewer the manuscript is long and complex to appeal to a broader range of readers. We shortened a bit the initial contents of the introduction and discussion by removing some details. However we believe there is a lot of very interesting points to mention in the discussion. Different people will be interested in different points of this discussion, in particular across species as well as across disciplines (geneticists versus physiologists). Therefore, we maintain all the discussion points while trying to shorten them.

Although we made an effort to shorten the manuscript, our cutting of sentences and restructuration had no impact on the final length of the draft.

First, as suggested by reviewer 1, we added more information to better follow the aim of each analysis and details on the variant calling method. We believe that by adding a short introduction paragraph to each method we have improved the clarity of the manuscript. We also add to include the Ethics statement at the end of the M&M section.

Second, a new section was also added in the discussion to answer the important ecological issues as suggested by reviewer 2.

Therefore we did not manage to shorten the manuscript at all. However, we believe the topic deserves this full length, and that we have really improve the clarity and the potential impact of the manuscript thanks to all reviewers’ comments.

2) Line 71: “primary sex determination”. Primary sexual differentiation sounds better to me.

The reviewer is right and this has been corrected: “During primary sex differentiation in vertebrates, […].”

3) Line 100: “follows” instead of “follow”?

Indeed, it has now been corrected.

4) Line 101: “testis or ovary” instead of “testis and ovary”?

Indeed, “and” has been changed to “or”.

5) Some abbreviations are defined more times (e.g., GSD). Some abbreviations do not have definitions (TGF-β, QTL).

We have corrected this error. GSD is now only defined once and we included the definition of: all abbreviations including those of cited genes.

6) Line 170: A period is missing behind a citation.

We corrected this typo and added a period after [34]. (line 174)

7) Line 612-613: If I follow this sentence well, it says that the gene gld-1 is localized in cytoplasm. It needs to be reformulated.

As we restructured the discussion to shorten it, we rephrase this sentence that now reads: “In nematodes, *gld*-1, the ortholog of *khdrbs* (KH domain-containing, RNA-binding, signal transduction-associated protein 2), is indispensable for oogenesis and meiotic prophase progression and it can stimulate sex determination of males in the hermaphrodite germ line [74, 75, 76].” (Lines 638-641)

8) Line 628: “fertile” is duplicated.

We removed the duplicated “fertile” (line 653).
